# Comparative Studies on the Organogel Formation of a Polyester in Three Different Base Oils by X-ray Analysis, Rheology and Infrared Spectroscopy

**DOI:** 10.3390/gels9090696

**Published:** 2023-08-29

**Authors:** Max Jopen, Michael Paulus, Christian Sternemann, Patrick Degen, Ralf Weberskirch

**Affiliations:** 1 Carl Bechem GmbH, Weststraße 120, 58089 Hagen, Germany; jopen@bechem.de (M.J.); degen@bechem.de (P.D.); 2 Fakultät Physik/DELTA, Technische Universität Dortmund, 44221 Dortmund, Germany; michael.paulus@tu-dortmund.de (M.P.); christian.sternemann@tu-dortmund.de (C.S.); 3 Fakultät für Chemie und Chemische Biologie, Technische Universität Dortmund, Otto-Hahn-Str. 6, 44227 Dortmund, Germany

**Keywords:** lubricants, polyester, thickener, organogel, small- and wide-angle X-ray scattering, rheology, FTIR

## Abstract

High-performance greases typically consist of a base oil and polyurea as a thickener material. To date, few alternatives to polyureas have been investigated. Polyesters could be one such alternative; however, little is known about the gelation of such polyesters because, unlike polyureas, they cannot form hydrogen bonds between the polymer chains. Here, we present studies on the gel formation of a polyester based thickener poly(hexane dodecanoate) with 1-octanol endgroups in three different base oils, i.e., a mineral oil (Brightstock 150), a synthetic Polyalphaolefin (Spectrasyn 40) and castor oil (85 to 90 wt.% ricinoleic acid triglyceride). Small- and wide-angle X-ray scattering measurements indicate a strong interaction of the polyester with castor oil and an increase in the crystalline fraction, with an increasing polymer amount from 5 to 40 wt.%. Moreover, infrared analysis of the polyester in castor oil showed gel formation at a minimum concentration of 20 wt.%. The strong interaction of the polyester with castor oil compared to the other two base oils led to an increase in the yield point γ_F_ as a measure of the mechanical stability of the gel, which was determined to be 5.9% compared to 0.8% and 1.0% in Brightstock and Spectrasyn, respectively.

## 1. Introduction

In industry, many processes are becoming increasingly automated, and as a result, the number of moving parts, such as bearings, gears or turbines is continuously increasing [1,2]. To make these parts as durable and efficient as possible, lubricants are used to reduce friction and wear [3]. The industry is faced with several challenges at the same time: While on the one hand, lubricants have to be developed for ever more demanding technical applications, the requirements for such developments in terms of sustainability and their environmental impact have continued to increase in recent years [4,5]. For example, many moving machine parts are operated via rolling bearings [6]. One general requirement for a lubricant suitable in such applications is a gel structure [7]; therefore, greases are usually used for this application. Lubricating greases usually consist of three components: the base oil (70–97%), the thickener (3–30%) and eventually, some additives (0–10%) [6]. While the base oil is already very often based on vegetable oil in commercial products [8,9], thickeners and additives are typically derived from petrochemical raw materials. The polymeric thickener in particular can account for up to 30 wt.% of the lubricant and is therefore a main component of a lubricating grease. Today, mainly oligourea-based thickeners are used in rolling bearings for high-performance applications [6]. The challenges with urea thickeners are two-fold: The starting materials are produced from petrochemical resources, and urea-based thickeners are not biodegradable and may be problematic for the environment. Previously, we have shown that it is possible to produce more sustainable lubricating greases based on urea thickeners by using renewable raw materials [10]. However, in the next stage of development of sustainable products, renewable raw materials could be combined with biodegradability for the polymeric thickener [11,12]. For example, more recently, a series of chemically modified biopolymers based on methylcellulose, chitin and cellulosic pulp were tribologically characterized and displayed negligible wear [13]. Despite the promising results, these biopolymers can only be varied in terms of their molecular weight and chemical composition to a very limited extent. In contrast, polyesters, for example, can be produced from renewable raw materials, are biodegradable, and can be built up in a very variable way in terms of molar mass and composition [11,12]. Interestingly, only few examples are known in the literature in which polyesters have been used as thickener materials in lubricants. They include xanthophylls-containing poly(3-caprolactone)s [14], azelaic acid based di- and polyester [15], polyhydroxyalkanoate based organogels [16,17], and a series of nine aliphatic polyesters that were tested for their thickening effect in lubricating greases [12]. Despite the successful application of polyester-based thickener materials, little is known to date about the structure of the gels formed.

Therefore, the aim of this work was to investigate the effect of different base oils on the organogel formation of the polyester thickener. For urea thickeners, it is known that the formation of the thickening structure takes place via the self-assembly of the thickener molecules or thickener chains [18]. A number of physical interactions play a role in this process; however, the most important one is hydrogen bonds [18,19] as the prerequisite of the urea units for self-assembly. For polyesters as a thickener, however, the formation of hydrogen bonds between the polymer chains is no longer possible since ester functional groups can only act as a hydrogen acceptor, not as a hydrogen donor. Moreover, when an attempt was made to dissolve two polyesters in thirty-six organic solvents, they were either insoluble or dissolved completely [20]. Gel formation was only observed when specific endgroups were attached to the polyester chain ends [20]. Therefore, it was of interest to us to better understand the mechanism of polymer aggregate formation in different base oils, especially for systems that are stable under load.

Here, we present the synthesis of a poly(hexane dodecanoate) **PT** with 1-octanol endgroups and a constant degree of polymerisation of nine by polycondensation from 1,12-dodecane diacid (DDS), 1,6-hexanediol (HD) and 1-octanol. Next, 25 wt.% thickener content was incorporated into three different base oils, Brightstock 150 (**BS150**, mineral oil), Spectrasyn 40 (**S40**, synthetic PAO oil) and castor oil (ricinoleic acid triglyceride), to investigate the influence of the base oil on the organogel-forming properties by X-ray scattering, infrared spectroscopy and rheological measurements. Secondly, **PT** was investigated in castor oil as a function of thickener concentration (5 wt.% to 40 wt.%) to determine the critical thickener concentration (CTC), i.e., the minimum amount of thickener above which gel formation occurs.

## 2. Results and Discussion

### 2.1. Gel Characterisation 

#### 2.1.1. Tube Inversion Test

**Figure 1:**
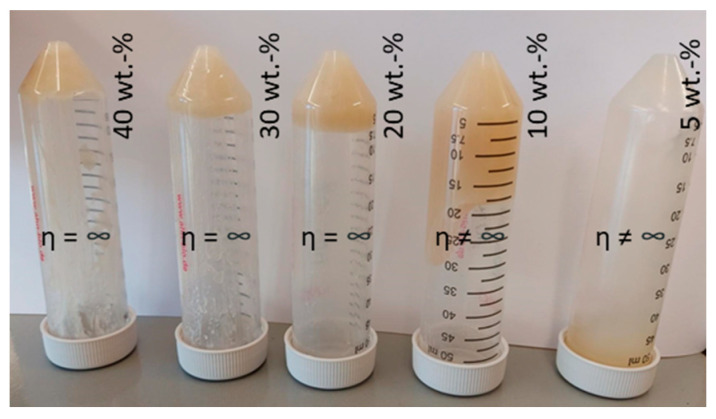
Results of tube inversion test for different amounts of **PT** in **CO**.

Tube inversion tests were performed as a first screening method to determine successful gel formation (Figure 1). Five samples of **PT** in **CO** were prepared with concentrations between 5 and 40 wt.%. We chose this concentration range because typical thickener content in a lubricant, e.g., based on polyurea, is between 5 wt.% and 20 wt.% [21]. The results showed that all samples above 20 wt.% **PT** passed the tube inversion test, whereas the samples with 10 wt.% or less **PT** showed a flow behaviour. Furthermore, all **PT** samples in the three different base oils—**BS150**, **S40** and **CO**—passed the tube inversion test at 25 wt.% thickener content. Several low molecular-weight gelators, which incorporate long *n*-alkyl chains, have already been described in the literature [22,23,24]. The long *n*-alkyl chains are supposed to help the association by van der Waals forces [25]. In comparison to the PT/base oil systems, the low molecular-weight gelators display a much lower minimum gelation concentration of 1–3 % (*w*/*v*). Gelation took place in more polar solvents such as propane-1-ol, propane-2-ol, butane-1-ol, butane-2-ol [25]. Polymeric gelators tend to have a higher minimum gelator concentration, more in the range of 2 to 10 % (*w*/*v*) [26,27] and sometimes even higher, with 10 to 40 % (*w*/*v*) [20].

#### 2.1.2. X-ray Scattering

**Figure 2:**
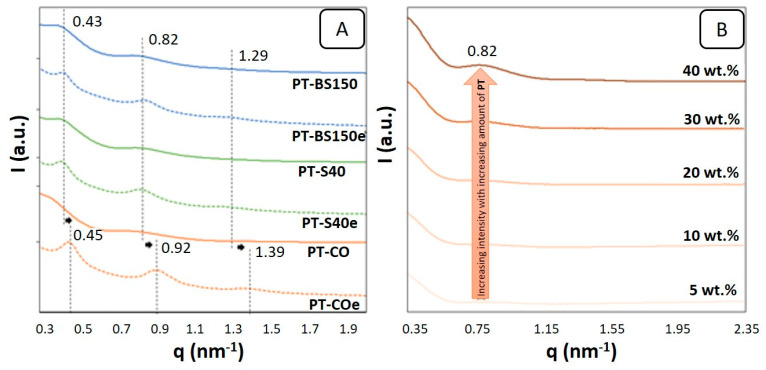
SAXS measurements for **PT** (25 wt.%) in different base oils **BS150**, **S40** and **CO** and extracted thickeners **e** (**A**), concentration series of **PT** in **CO** (**B**).

**Figure 3:**
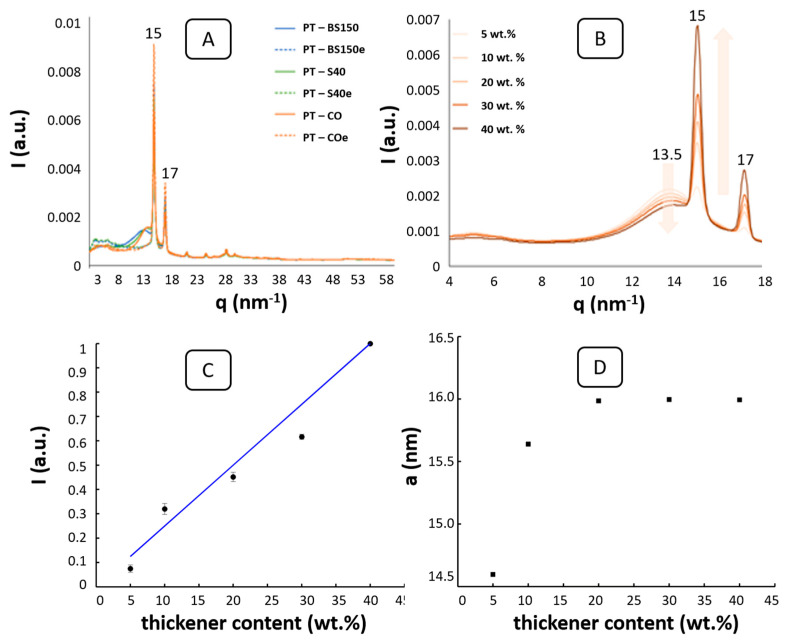
WAXS measurements for **PT** in different base oils **BS150**, **S40** and **CO** and extracted thickeners **e** (**A**), concentration series of **PT** in **CO** (**B**), intensity of the scattering signal of the crystalline phase as a function of the thickener concentration for the **PT-CO** system (**C**) (the intensity is normalized on the intensity of the 40 wt.% data), and length of the *c*-axis as a function of the thickener concentration (**D**).

In the first set of experiments, greases based on 25 wt.% **PT** in a base oil were analysed and compared to the polyester **PT** after extraction from the base oil. When inspecting the spectra of the extracted thickeners and that of the thickeners after synthesis, it is noticeable that the extraction process has a marked effect on the position of the Bragg reflection in the case of **CO**, while much smaller shifts occur in the case of the other oils (Figure 2A). This effect is attributed to the increased interaction between the CO and the thickener, which causes a shortening of the unit cell during the extraction process. The differences in peak heights and widths are striking when the spectra of the three different fats are considered. Here, the reflections of the system with **CO** appear less pronounced and broader than those of the oil–polyester mixtures in **S40** and **BS150**. This can be explained by the increased solubility of **PT** in **CO**, which would involve a general decrease in the crystalline fraction and the size of the crystalline domains. The X-ray scattering data of the **PT** system shown in Figure 2 can be described by a triclinic crystal system whose unit cell has significantly different lengths depending on orientation. Here, the Small-angle X-ray scattering (SAXS) data in Figure 2A,B show maxima that point to an extension in the (00l) direction on the order of 15.5 nm, while the (Wide angle X-ray scattering) WAXS data (Figure 3A,B) show Bragg reflections that fix the lateral extension ((hk0) directions) at about 0.37 nm and 0.42 nm, respectively (see Figure 3). The angle enclosed by the lateral unit vectors is 83°. It is notable that the extracted thickener from the **PT/CO** mixture indicates a decrease in (00l) direction. All SAXS maxima of the extracted thickeners decrease in width, accompanied by an increase in intensity.

In addition to the Bragg reflections, the WAXS data of the polyester systems show the well-known first sharp diffraction peak (FSDP) at about 13.5 nm^−1^, which is characteristic for liquids and amorphous systems referring to typical particle–particle distances of about 4.6 Å in the oils and, hence, is absent in the extracted thickeners. Moreover, we recognize a so-called pre-peak in the case of the polar **CO** at about 5 nm^−1^, related to a typical distance of 12.6 Å in contrast to the non-polar **BS150** and **S40,** indicating a heterogeneous microstructure of **CO**. This is often observed in associated liquids and can be related to the formation of transient supramolecular arrangements characterised by the interplay between hydrogen-bonding and the steric hindrance of the non-polar parts of the molecules (see e.g., [28,29,30] and references therein). When varying the oil fractions, the WAXS spectra (Figure 3B) do not show any significant changes in the crystal structure. Only the relative scattering contributions of the oil phase and the thickener shift against each other.

The SAXS measurements (Figure 2B) as a function of the portion of **PT** showed an increase in the maximum at 0.8 nm^−1^ with increasing thickener proportion. This increase directly reflects the increasing amount of crystalline thickener in the sample and is confirmed by the decrease in the FSDP observed in the WAXS data presented in Figure 3B. The increase in the crystalline fraction was examined in more detail by integrating the Bragg reflections (see Figure 3C), displaying a linear increase in the crystalline fraction with the thickener concentration. Furthermore, by analysing the SAXS spectra, the length of the **PT**’s *c*-axis in the system was determined as a function of the thickener concentration (Figure 3D). This value increases with increasing thickener concentration and reaches a plateau of approx. 16 nm at 20 wt.% and above. At concentrations below 20 wt.% thickener content, the **CO-PT** interaction is critical as it affects the **PT**’s crystallinity and can be seen with the broader peaks of PT as well. At higher **PT** concentrations, the growing crystalline **PT** regions are hardly affected by **CO** anymore and reach a critical size.

#### 2.1.3. Infrared Spectroscopy (IR)

**Figure 4:**
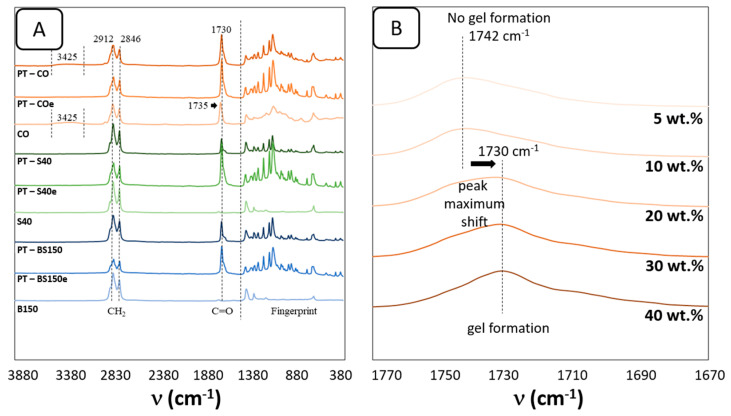
Results of IR spectroscopy for **PT** in **BS150**, **S40** and **CO**, pure base oils and extracted thickeners **e** (**A**), concentration series of **PT** in **CO** (**B**).

The characteristic absorption bands for carbonyl-stretching vibrations (C=O, 1740 cm^−1^) and the C–O–C stretching (1153 cm^−1^) of the ester groups in the polymer backbone confirm the successful formation of a polyester. However, as can be seen the polymeric thickener **PT** showed no significant differences in the IR absorption spectra both in the different oils and after extraction from these oils (Figure 4A).

The concentration-dependent transition from a viscous liquid to a three-dimensional gel structure can often be followed by a shift in the maxima of the IR signals involved. For polyureas, this can be observed for N-H-induced hydrogen bonding between the thickener chains in the range 3275–3500 cm^−1^ [18,19,31,32,33,34]. Figure 4B shows concentration-dependent differences in the absorption maximum of the ester C=O group. For concentrations of 5 and 10 wt.%, the peak maximum is at 1742 cm^−1^, whereas at 20 wt.% and above, the maxima shift to 1730 cm^−1^. This is in agreement with the results from SAXS measurements. As long as the **CO–PT** interaction is dominant at a **PT** concentration below 20 wt.%, there is no gel formation. Due to the superposition of the ester bands of the oil and the thickener network, intensities for both bands are always recognisable, independent of the thickener concentration. The only decisive factor here is the position of the local maximum. As long as the local maximum is higher at 1742 cm^−1^, the properties of the oil dominate over the physical thickener network. When comparing the IR spectra at 10 and 20 wt.% thickeners, it can be seen that both maxima show a similar intensity. In comparison, when gels based on poly(3-hydroxybutyrate) were analysed by IR, a positive peak at 1743 cm^−1^ was observed which corresponds to the C=O stretching of PHB in the amorphous state. Moreover, it was observed that when PHB forms intermolecular hydrogen bonds during the gelation process, its IR spectra give rise to a characteristic C=O stretch band at 1722 cm^−1^ [16].

Polymeric gelators based on a polyester terminated with alkylated L-lysine residues on both ends showed an IR band ν(C=O) of the ester group at 1730 cm^−1^, independent of their state, i.e., as a gel or in solution, wherein it was interpretated that no intermolecular interactions were involved [20].

The band at 3425 cm^−1^, which is indicative of the hydrogen bonding of CO groups, suggests a stronger interaction of the castor oil and the polyester C==O bonds, which was not observed below 20 wt.% PT. IR bands at 2912 and 2846 cm^−1^ arise from anti-symmetrical and symmetric stretching vibrations of C-H bonds, indicative of closely packed alkyl chains that form a crystalline domain [25,35].

#### 2.1.4. Rheology

**Figure 5:**
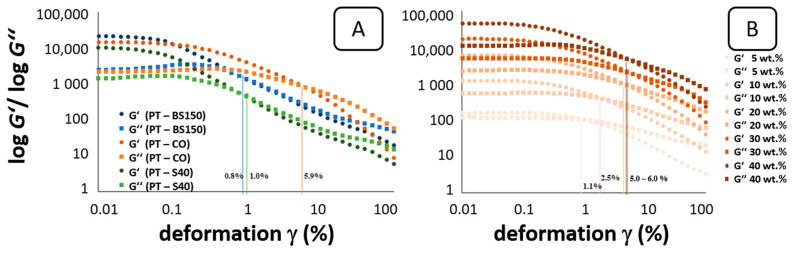
Results of rheological measurements for **PT** different base oils **BS150**, **S40** and **CO** (**A**) and concentration series of **PT** in **CO** (**B**).

To characterize the mechanical behaviour of the greases, we performed rheological tests for different base oils (**BS150, S40** and **CO**) and different thickener concentrations for one base oil (CO). First, dynamic strain sweep tests were performed in order to investigate the linear viscoelastic region (LVE-region) and the flow limit (mechanic stability) γ_F_ of the grease systems **PT-BS150, PT-S40** and **PT-CO** (Figure 5A). The LVE region can be determined by the maximum strain of the plateau regime, where the elastic (*G*′) and viscous (*G*″) modulus are constant. If the deformation increases further, a decrease in the elastic modulus *G*′ can be observed, indicating the loss of the LVE region and the partial destruction of the network structure. Regarding the intersection point between storage (*G*′) and loss modulus (*G*″), the strain sweep experiments allow the determination of the flow limit γ_F_.

It was previously shown by X-ray scattering that the thickener shows larger-length scales for the superstructure of the physical network, i.e., larger crystalline domains in the two non-polar base oils compared to the polar castor oil. This could be attributed to a higher crystalline content of the thickener due to the poorer solubility of PT in these base oils. For the systems in **BS150** and **S40**, smaller values for γ_F_ than in **CO** were therefore expected due to lower interactions with the base oil. In **CO**, γ_F_ was 5.9%, whereas in **BS150** and **S40**, γ_F_ was determined at 0.8% and 1.0%, respectively. The results correlate with the observations in the X-ray scattering. Overall, γ_F_ for the non-polar oils is very low, at about 1%. The two greases thus show a flow behaviour with almost no mechanical stress. This is due to the lack of hydrogen bonding between the oil and the thickener. The stability of these systems without mechanical stress would thus be dominated purely by the semi-crystallinity of the thickener. With decreasing numbers of CH_2_ groups and thus a decreasing crystalline fraction [11], the formation of gel-like structures is thus no longer to be expected. In castor oil, γ_F_ was also investigated as a function of thickener concentration (Figure 5B). It is well known that the relative flow limit γ_F_ in % is independent of the thickener content [36]. However, below the CTC, a deviating behaviour is thus to be expected since samples below the CTC already show an independent flow behaviour without mechanical stress. The results of the IR-spectroscopy investigation showed that the CTC is below 20 wt.% for the **PT**-**CO** system; thus, changes for γ_F_ should only be observed at 5 and 10 wt.%.

As can be seen in Figure 5B, the determined *γ_F_* values were at 5.0–6.0% when samples with 20 wt.% to 40 wt.% **PT** were employed. The deviations were within the error range of the device. In contrast, at lower thickener concentrations of 5 and 10 wt.%, *γ_F_* values were 1.1% and 2.5%, respectively, indicating clearly the transition from a gel-like structure to a viscous fluid, in agreement with the finding from the IR spectroscopy below (20 wt.%). For Newtonian fluids, no visible flow limits would have been expected [37]. Below the CTC, the **PT** system thus shows the behaviour of a viscoelastic fluid [37]. In comparison, the flow limit *γ_F_* of various polyurea-based greases, such as MDI-ODA, MDI-HDA and MDI-MDA, with stearylamine endgroups in PAO6 is significantly higher, being 69%, 65% and 37%, respectively [38].

**Figure 6:**
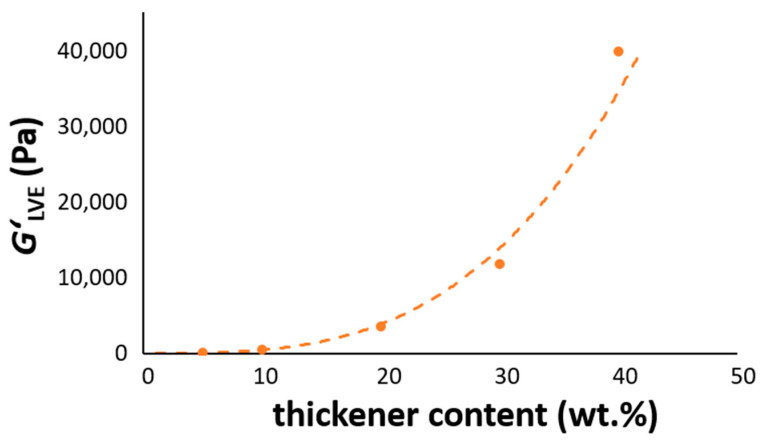
*G*’ at the LVE level as a function of **PT** concentration in **CO** from 5 to 40 wt.%.

In Figure 6, we compare the *G*′ values at the LVE region and the evolution of crystallinity as a function of the thickener concentration. The *G*′ values can be derived from the amplitude tests (Figure 5B). We can clearly see that both the *G*′ values and the amount of crystallinity increase when the concentration of the thickener is increased. Surprisingly, the increase in the *G*′ values is exponential, whereas the crystallinity shows only a linear dependence on the thickener concentration. This can be explained by the fact that in addition to crystalline domains, there are also amorphous cross-linking points in the grease structure. In addition, the results indicate that these amorphous crosslinking points increase in importance with increasing thickener concentration. Compared to aliphatic polyurea thickener in castor oil, the **PT** system has a comparatively lower flow limit of 5.0–6.0% [10]. However, since the overall flow limit is dependent on the chain mobility [10], and since this is limited by the higher crystallinity of the **PT** system [11], it may be possible to increase the flow limit by using shorter aliphatic dicarboxylic acids. Whether this is necessary or desirable depends on the respective application.

**Figure 7:**
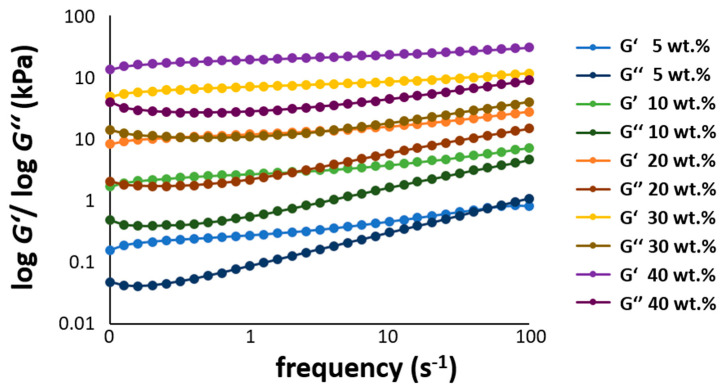
Frequency sweep experiments for a series of **PT** in **CO** from 5 to 40 wt.%.

Finally, we performed frequency sweep experiments that describe the time-dependent behaviour of a sample in the non-destructive deformation range. We chose a deformation of 0.1% to make sure that the tests were performed in the LVE region of the grease (Figure 7). High frequencies are used to simulate fast motion on short timescales, whereas low frequencies simulate slow motion on long timescales or at rest. In practice, frequency sweeps are proven methods for gathering information on the behaviour and inner structure of gel-like structures.

The curves show predominantly elastic behaviour at low frequencies. Especially at high frequencies, however, there is a clear difference at different concentrations. For 5 wt.% and 10 wt.% thickener contents, the samples show a yield point at high frequencies. For 5%, this gel–sol transition is actually measured; for 10%, we extrapolated it at approx. 130 rad/s. These two samples behave like rubbers at the boundary between rubber elastic behaviour and the transition area to the glass point.

## 3. Conclusions

The aim of our work was to investigate the organogel formation of a poly(hexane dodecanoate) **PT** with 1-octanol endgroups in three base oils, Brightstock 150 (mineral oil), Spectrasyn 40 (synthetic PAO oil) and castor oil (bio-based ester oil). Analysis by SAXS and WAXS measurements of **PT** displayed a significant structural difference in the polymer network and how the polyester interacts with the respective base oils. X-ray scattering shows the formation of larger crystalline domains in the two non-polar base oils compared to the polar castor oil. This could be attributed to the poorer solubility of **PT** in these base oils. The difference in polyester-base oil interactions could also be detected by rheology measurements and the flow point γ_F_ of 5.9% for the polyester-castor oil system, whereas in Brightstock 150 and Spectrasyn 40, γ_F_ was determined at 0.8% and 1.0%, respectively. Moreover, a concentration-dependent analysis of the polyester in castor oil indicated a transition from a viscous liquid to a gel-like structure at around 20 wt.%, as can be seen by the IR analysis and rheology experiments. Our results provide detailed insights into the solvent-dependent gelation of the polyester thickener and should be helpful for the development of novel thickener-based oil lubricant systems.

## 4. Materials and Methods

### 4.1. Materials

Castor oil from Carl Roth (Karlsruhe, Germany), 1,12-dodecane diacid from TCI (Tokio, Japan), 1,6-hexanediol from Sigma-Aldrich (St. Louis, MO, USA), *p*-toluenesulfonic acid from Sigma-Aldrich (St. Louis, USA) and 1-octanol from TCI (Tokio, Japan) were used to produce the polyester greases.

### 4.2. Polyester Synthesis

**Scheme 1:**
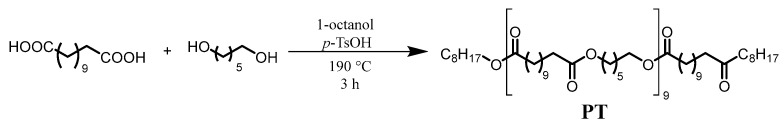
Synthesis of polymeric thickener **PT** from 1,6-hexanediol,1,12-dodecanoic acid and 1-octanol.

**PT** was produced by melt polycondensation for 3 h at 190 °C without solvent. As catalyst, *p*-toluenesulfonic acid (200 mg) was used. Therefore, 1,12-dodecanedioic acid (9 g, 1 eq.), 1,6-hexanediol (0.75 eq.) and 1-octanol (0.25 eq.) were placed in a 500 mL three-neck flask, heated to 190 °C, and the resulting melt was stirred at 500 rpm (Scheme 1) [12]. After 3 h, the reaction mixture was cooled to 160 °C, divided into several parts, and the base oil was added. Three different base oils were used for these experiments: **BS150**, **S40** and castor oil (**CO**). Stirring was continued at 160 °C for another 10 min. The reaction mixtures were then allowed to cool to room temperature while stirring.

After cooling to room temperature, the greases were homogenized by means of a roller. A theoretical value of 9 was assumed for the degree of polymerization, calculated using the Carothers equation [39] and confirmed by ^1^H-NMR spectroscopy (see Figure S1). Sample preparation was performed in the same way for all base oils.

$$P_{n}=\frac{1+r}{1+r-2rx}\;\mathrm{with}\;r=\frac{N_{A0}}{N_{B0}+2N_{B^{\prime }0}}$$

with *x* = conversion, *N_A_*_0_ = diacid, *N_B_*_0_ = dialcohol, *N_B′_*_0_ = monoalcohol.

### 4.3. Tube Inversion Test

The tube inversion test is used as an indicator for successful gelation. Here, a closed vessel, e.g., a 50 mL Falcon^TM^ tube, is turned upside down with the sample. If the sample flows during the test, the sample has not gelled and therefore failed the test. The tube inversion test was performed as described in the literature [40].

### 4.4. X-ray Scattering

To obtain information on the structural composition of the lubricants, small-angle X-ray scattering (SAXS) and wide-angle X-ray scattering (WAXS) were performed at the DELTA synchrotron radiation source. SAXS data were acquired at beamline BL2 with a photon energy of 12 keV, a beam cross section of 0.5 × 0.5 mm^2^, and a sample-detector distance of 1400 mm. A silver behenate sample was used to calibrate the setup. The WAXS measurements were recorded at beamline BL9 [41] with a photon energy of 27 keV. The distance between the sample and the detector was 337 mm. The beam size was 1.5 × 1 mm^2^ (horizontal × vertical). A silicon powder sample was used to calibrate the setup. All measurements were recorded at a temperature of 25 °C. The samples were placed in a 1.5 mm thick sample holder. A MAR345 image plate detector was used for data collection at both beamlines. The evaluation of the data was conducted with the software fit2d [42].

### 4.5. Infrared Spectroscopy

The IR spectra were measured on a Tensor 27 Platinum Diamond ATR instrument (Bruker, Billerica, MA, USA). The spectra were recorded by 32 scans and with a standard wave number range of 600–4000 cm^−1^ at a resolution of 4 cm^−1^. The data were plotted reciprocally. Data processing was conducted with Opus 7.0.

### 4.6. Rheology

Oscillation measurements were performed on an MCR 102 rheometer (Anton Paar, Ostfildern, Germany). All measurements were carried out at 25 °C. Data processing was conducted with RheoCompass 1.3. A plate–plate geometry with a diameter of 25 mm was used with a shear deformation of 0.01% to 100% (41 data points), constant angular frequency of 10 rad/s, and constant gap size of 1.000 mm. For trimming, a gap size of 1.025 mm was used.

Two types of measurements were performed for the rheological characterization of the grease. A frequency-sweep test performed with a constant strain (γ = 1.0%) gave detailed information about the network properties, while a strain sweep test at constant frequency (ω = 1 rad/s) allowed for the evaluation of the stability of the polymer network. Throughout these experiments, it was possible to determine the degree of cross-linking, the relaxation properties, the linear viscoelastic region (LVE-region) of the polymer gel, and the flow limit γ_F_ of the grease (according to DIN 51810-2).

## Data Availability

Not applicable.

## Supplementary Materials

The following supporting information can be downloaded at https://www.mdpi.com/article/10.3390/gels9090696/s1: Figure S1: ^1^H NMR of **PT** after synthesis for base oil experiments; Figure S2: ^1^H NMR of **PT** after synthesis for concentration series of **PT** in castor oil (**CO**); Figure S3: SAXS measurements for **PT** in concentration series of **PT** in **CO**; Figure S4: SAXS measurements for **PT** in different base oils **BS150**, **S40** and **CO** and extracted thickenerse.
