# Supplementary material for: Comparative Studies on the Organogel Formation of a Polyester in Three Different Base Oils by X-ray Analysis, Rheology and Infrared Spectroscopy
Source: Gels. 2023 Aug 29;9(9):696. doi: 10.3390/gels9090696 (PMC10530540; doi:10.3390/gels9090696)
Supplement: Supplementary file 1 [file gels-09-00696-s001.zip › gels-2539229-supplementary.pdf]

Supporting Information

Comparative Studies on the Organogel Formation of a Polyester in Three Different Base Oils by X-ray Analysis, Rheology and Infrared Spectroscopy

Max Jopen [1], Michael Paulus [2], Christian Sternemann [2], Patrick Degen [1] and Ralf Weberskirch [3,*]

[1] Carl Bechem GmbH, Weststraße 120, 58089 Hagen, Germany; jopen@bechem.de (M.J.); degen@bechem.de (P.D.)
[2] Fakultät Physik/DELTA, Technische Universität Dortmund, 44221 Dortmund, Germany; michael.paulus@tu-dortmund.de (M.P.); christian.sternemann@tu-dortmund.de (C.S.)
[3] Fakultät für Chemie und Chemische Biologie, Technische Universität Dortmund, Otto-Hahn-Str. 6, 44227 Dortmund, Germany
* Correspondence: ralf.weberskirch@tu-dortmund.de

Content	page
1. ^{1}H NMR data of polyester thickener (**PT**) after synthesis; Fig. S1- S2	2
2. SAXS measurement data of **PT**, Fig. S3-S4	3

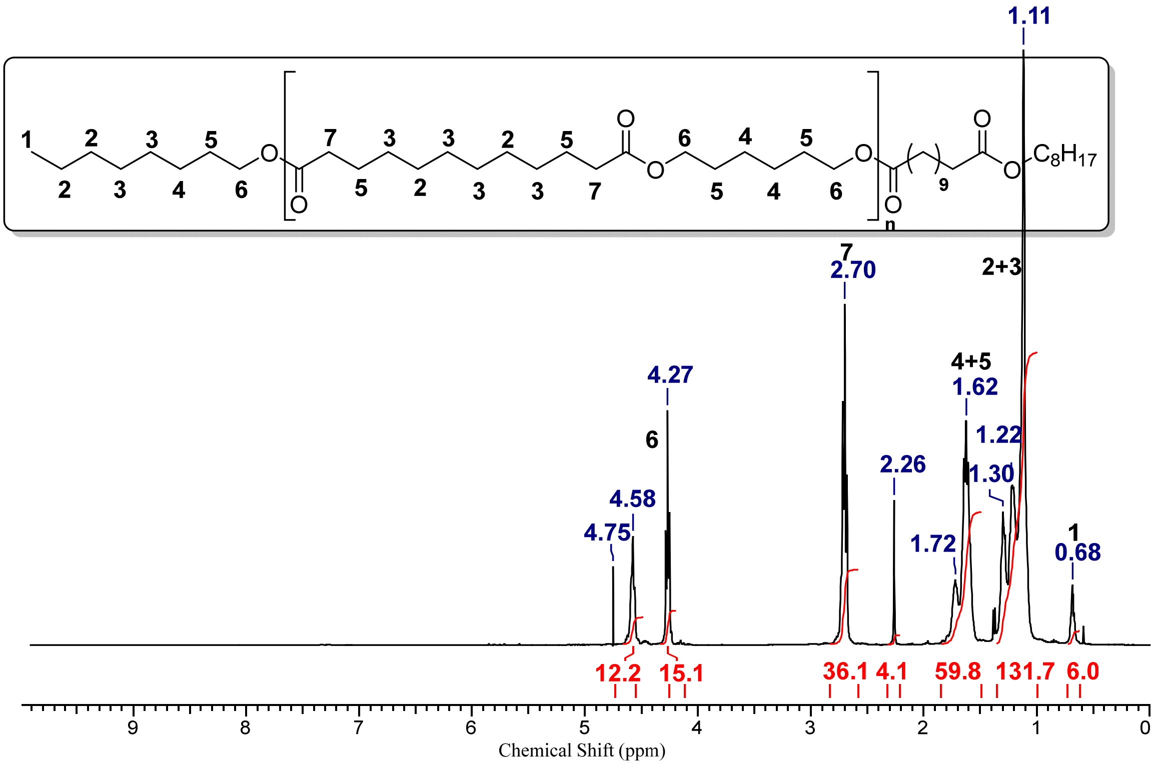

Figure S1: ^{1}H NMR of **PT** after synthesis for base oil experiments.

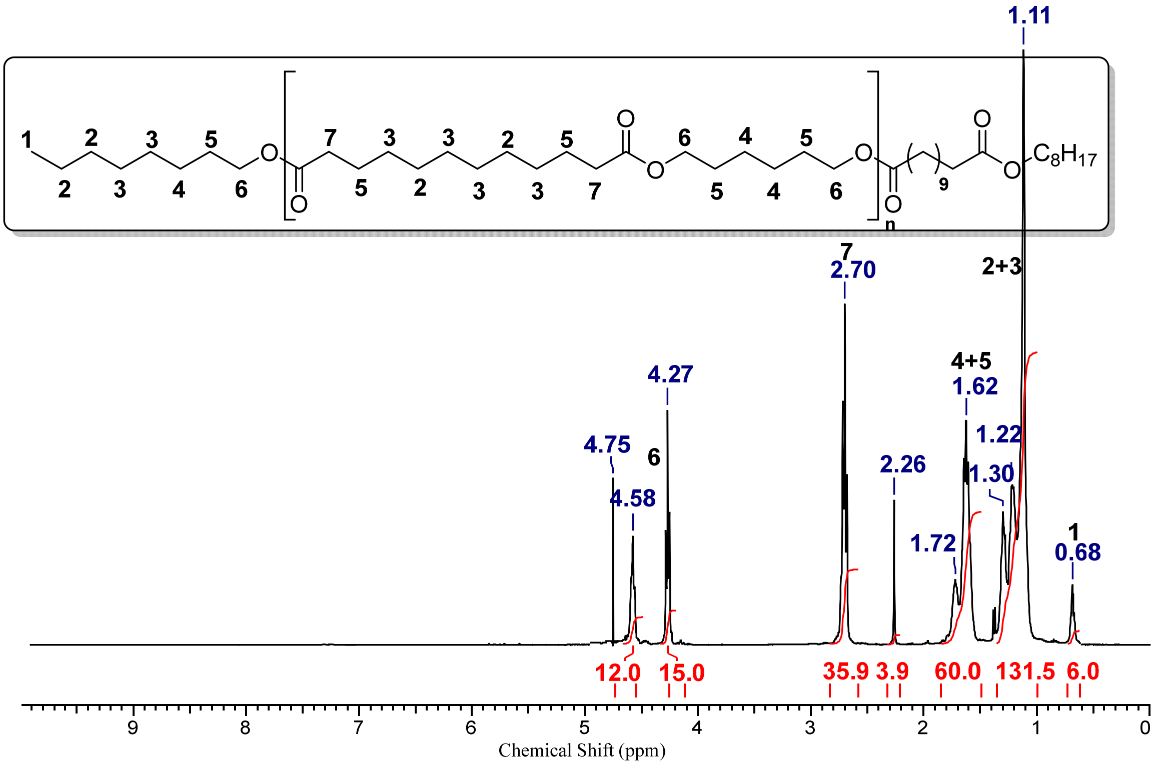

Figure S2: ^{1}H NMR of **PT** after synthesis for concentration series of **PT** in castor oil (**CO**).

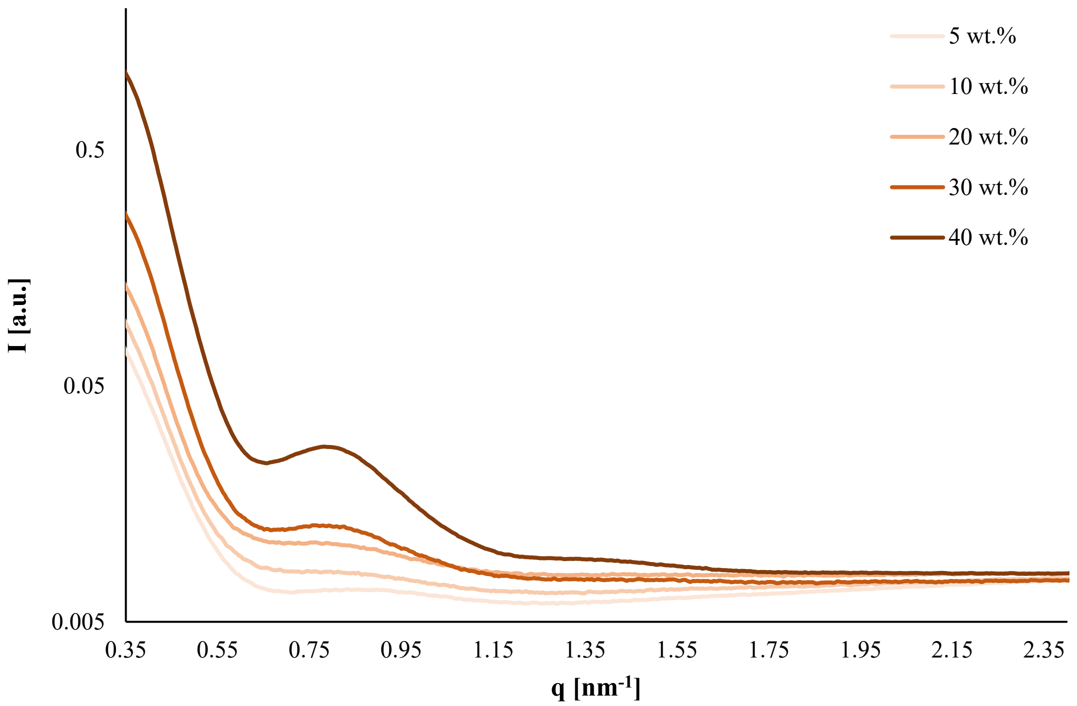

Figure S3: SAXS measurements for **PT** in concentration series of **PT** in **CO**.

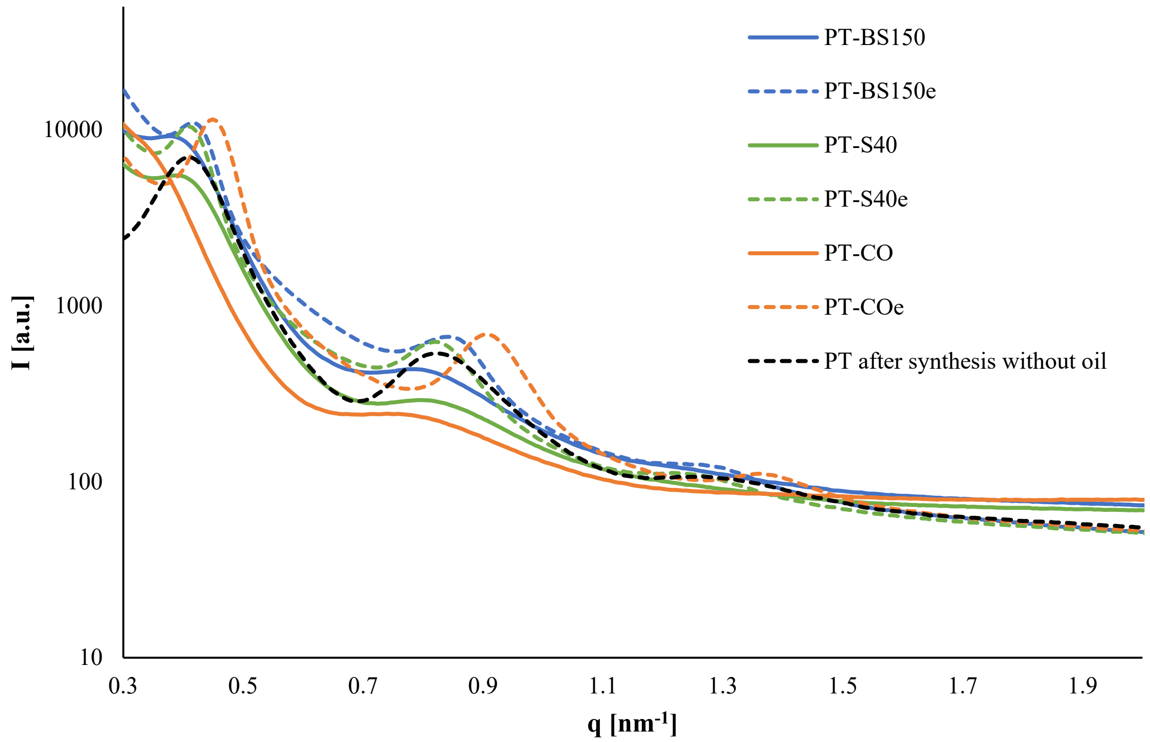

Figure S4: SAXS measurements for **PT** in different base oils **BS150**, **S40** and **CO** and extracted thickeners **e**.
